# Supplementary material for: Are carbon emissions trading and green financial instruments synergistic? -Comprehensive quantitative research based on content analysis
Source: PLoS One. 2024 Mar 7;19(3):e0298601. doi: 10.1371/journal.pone.0298601 (PMC10919601; doi:10.1371/journal.pone.0298601)
Supplement: S1 Data — (ZIP) [file pone.0298601.s001.zip › Data/地级市Z_F工作报告.zip!/╜¡╦╒/╦▐╟¿╩╨/└┤╫╘╣1⁄2╓┌║┼í╛┬φ┐╦╩2╛▌═°í┐_2012.docx]

各位代表：

现在，我代表宿迁市人民政府，向大会作工作报告，请予审议，并请各位政协委员和其他列席人员提出意见。

“十二五”取得良好开局

2011年，是“十二五”的开局之年，也是全市上下攻坚克难、奋发有为的一年。在省委、省政府和市委的坚强领导下，在市人大和市政协的监督支持下，我们紧紧围绕“推进更大突破、实现全面小康”的奋斗目标，抢抓机遇，迎难而上，较好地完成了市三届人大四次会议确定的各项目标任务，实现了“十二五”良好开局。主要指标取得重大突破，预计完成地区生产总值1309亿元，增长12.8%，人均达2.76万元、突破4000美元；实现财政总收入276.2亿元，增长34.3%；一般预算收入首次登上百亿元台阶、达121亿元，增长35.1%。发展速度保持全省领先，财政一般预算收入、规模以上固定资产投资、规模以上工业增加值、进出口总额、存贷款、城镇居民人均可支配收入、农民人均纯收入等指标增速继续位居全省前列。民生投入实现大幅增长，各级财政用于民生投入达125.4亿元，增长30.4%，占财政一般预算支出的57.9%。

一年来，我们主要做了以下几个方面的工作：

一是科学应对宏观形势，经济运行平稳健康。面对复杂多变的宏观环境，及时采取针对性措施，推动经济平稳较快增长。工业经济量质齐升。全面落实扶持政策，积极化解要素制约，规模以上企业净增251户，累计达1773户，完成增加值372亿元，增长21.2%。酿酒食品、纺织服装、林木加工、机械电子四大百亿级产业累计实现销售收入870亿元，增长45%。规模以上新兴产业销售收入达312亿元，增长41.8%。智能电网等5个园区获批省级特色产业基地（园）。苏酒集团主营业务收入稳居白酒行业前三强。“梦之蓝”、“玖久”、“SPCC”获批中国驰名商标，累计达10件，总数位居苏北第二。质量兴市先进市顺利通过省级验收。农业生产再创佳绩。大力推进农业现代化建设，新增高效农业45.4万亩、渔业15.9万亩。实施水稻“四改”工程，粮食产量实现“八连增”。三县两区现代农业产业园全部获批省级园区，运河湾现代农业产业园获批国家农业产业化示范基地。苏北花卉、金水集团被认定为国家级农业龙头企业。服务业发展势头强劲。深入实施服务业提速计划，服务业实现增加值494亿元，增长12.1%。旅游业加速突破“三年行动计划”扎实推进。市软件园获批省国际服务外包人才培训基地和两化融合服务产业示范园。省第七届园艺博览会、省第四届森林生态旅游节成功举办，得到省委省政府的充分肯定和社会各界的广泛赞誉。

二是突出转型升级主线，结构调整步伐加快。产业结构不断优化，三次产业比例调整为15.8:46.2:38，二三产业占比提高1.8个百分点。增长动力持续增强。持之以恒加大投入，完成规模以上固定资产投资764.4亿元，增长24.6%。组建市直八个专业招商局，举办中国（宿迁）首届经贸洽谈会、省属企业洽谈会，赴苏州、南通、厦门、香港、台湾等地举办招商活动，新引进亿元以上合同项目339个、新开工111个、新竣工84个。实现社会消费品零售总额338.6亿元，增长18.1%。完成进出口18.8亿美元，增长54.1%，其中出口15.3亿美元，增长66.3%。宿迁国检挂牌成立。南北挂钩、共建园区工作走在全省前列。融资水平逐步提高。金融机构存贷款余额分别达998亿元、760亿元，增长23%和22.2%。秀强股份、双星新材成功上市。农村小贷公司新设立10家，累计达41家，总数位居苏北第一。农合行全部改制为农商行。创新能力显著提升。首届宿迁生态科技博览会暨产学研合作洽谈会成功举办，东南大学（宿迁）产业技术研究院等5个科技平台挂牌运行。高新技术企业新增14户，累计达29户，实现产值161亿元，增长88.3%。我市被科技部表彰为“全国科技进步先进市”。创业创新领军人才“千人计划”正式启动。节能减排扎实推进。采取有保有压措施，控制高耗能产业发展，加快淘汰落后产能，实施重点节能和循环经济项目40个。新增污水日处理能力16万吨，主要污染物年度减排任务基本完成。

三是加快重点工程建设，城乡面貌变化显著。城镇功能持续提升。洋河新城、骆马湖省级旅游度假区正式挂牌。古黄河水景公园、生态体育公园建成开放，宝龙城市广场、金鹰国际购物中心、星辰国际酒店、华东农业大市场等开业运营，全民健身中心、游泳馆等体育场馆建成使用，宿迁1897、雨润广场等城市综合体建设稳步推进，运河文化城、项王故里等旅游项目开工建设。沭阳蓝天国际商贸城、泗阳雨润新天地广场、泗洪富园广场等加快推进。截污导流工程投入使用，市区第二水厂并网供水，七堡引水枢纽工程管道贯通，西民便河等灾后治理工程按计划实施。小城镇新增住房2.85万套，农民集中居住区新建住房3.28万套。城乡环境逐步优化。顺利通过省级卫生城市验收。启动国家级生态市和全国绿化模范城市创建，建成8个省级生态乡镇、9个省级生态村和26个市级生态村，全市森林覆盖率达28.02%，中心城市绿化覆盖率、绿地率分别达40.92%和37.1%。精品城市创建取得新成效，城市管理水平显著提升。积极开展“美好宿迁建设行动”，农村环境综合整治深入推进，疏浚县乡河道912公里，整治河塘1031处，创成省康居示范村4个、省人居环境范例奖1个。交通体系不断完善。大力推进交通重点工程建设，宿淮铁路、宿新高速、205国道宿迁段等扎实推进，326省道、330省道等启动建设，京杭运河宿迁大桥建成通车，“虚拟高铁站”正式运营。新增城市公交车101辆、更新52辆。全市新建农村公路212公里，行政村客运班车通达率达91%。

四是推进民生实事项目，人民生活持续改善。高度重视民计民生，扎实推进民生幸福工程建设。启动实施居民收入“六年倍增”计划，城镇居民人均可支配收入、农民人均纯收入分别达14926元、8315元，增长17%和19.2%。新发展私营企业1.14万户、个体工商户6.38万户。新增城镇就业4.1万人，新转移农村劳动力9万人，援助困难群体就业4900人，失业率控制在3%以内，动态消除城镇零就业、农村零转移家庭。社会保障水平不断提高，城镇职工“五险”扩面20.8万人次，新农保扩面接续184.3万人，城乡低保标准提高到每人每月260元、210元，农村五保集中和分散供养标准提高到每人每年3500元、2800元，城镇居民医保和新农合筹资标准提高到年人均270元、230元。建立物价上涨补贴联动机制，发放物价补贴5500万元，受益群众达26万人。新开工经济适用房6688套，新增廉租房1048套、公租房9968套，建设拆迁安置房213.3万平方米，保障性住房建设超额完成省定任务。启动实施西南岗新一轮整体帮扶，研究部署成子湖周边地区集中连片开发，提前完成省定脱贫攻坚任务。

五是提升公共服务功能，各项事业协调发展。牢固树立“科教优先”战略，加快教育现代化建设。培育优质教育资源，组建教育集团34个。省优质幼儿园比例达56.3%，初中毕业生升学率达98.3%，省三星级以上高中、职业学校比例分别达56.3%、65%，高考本科达线率首次达到全省平均线。校安工程开工量、竣工量位居全省前列。留守儿童教育管理工作得到国务院领导肯定。深化医药卫生体制改革，探索以“补需方”为原则对接基本药物制度。积极筹建市第一人民医院，市中医院获批三乙医院。提高城乡公共卫生服务和医疗服务水平，农村三级卫生服务网络基本形成，城市社区卫生服务中心覆盖率达100%，人均基本公共卫生服务专项资金提高到25元。启动实施文化强市“三步走”行动方略，公共文化场馆全部免费开放，新增农村有线电视用户10.9万户，公共文化服务水平不断提高。第六届宿迁创业文化节成果显著，我市被评为全国文化体制改革先进地区。全民健身活动广泛开展，市第三届运动会、亚洲小轮车锦标赛等赛事活动成功举办。人口计生管理与服务水平不断提升，人口自然增长率控制在5‰以内。积极构建“社会保障、社会平安、群众问政”三大网络，社会管理创新深入推进。加强城乡社区建设，小区物业实现属地化管理，“六进”小区工作稳步开展。应急管理工作水平和突发事件应急能力显著提升。全面加强校车安全管理，强化食品药品安全监管，安全生产形势得到进一步巩固。认真做好国防动员、优抚安置工作，“双拥”创建扎实推进。民防、民族宗教、外事侨务、对台事务、档案、史志工作取得新进展，妇女、儿童、青少年、老龄、残疾人、红十字、慈善事业取得新进步。扎实推进平安宿迁、法治宿迁建设，公众安全感位居全省第一，社会大局保持和谐稳定。

六是加强政府自身建设，发展环境明显优化。把抓落实作为政府工作的生命线，发扬苦干实干快干作风，落实巧干会干善干举措，坚持对政府工作每周一会商、重点项目每月一会办，确保工作环环相扣、项项落实。建立健全市区长联席会议制度，形成推动中心城市加快发展的长效机制。深入开展领导干部下基层“三解三促”活动，真心实意帮助群众解难事、办实事。综合性行政服务平台加快建设，行政权力网上公开透明运行扎实推进。“网上宿迁”在全国近300个地市级政府网站评比中进入前十强，“网络问政”栏目荣获“用户满意奖”。依托市行政学院，设立市情研究中心，加强对重大问题的咨询和研究，促进决策的民主化与科学化。强化法治政府建设，行政复议、出庭应诉制度不断完善，依法行政得到进一步加强。自觉接受市人大及其常委会法律监督和市政协民主监督，认真执行人大及其常委会决议，积极支持政协履行职能，高度重视人大代表议案、建议和政协委员提案办理工作。严格执行党风廉政建设责任制，强化经济责任审计，推行行政效能监察，领导干部廉洁自律意识进一步增强。

各位代表，回顾过去的一年，成绩来之不易，奋斗充满艰辛。我们深深体会到，宿迁发展取得的每一点成绩、城乡面貌发生的每一点变化、广大群众得到的每一点实惠，都离不开省委、省政府和市委的正确领导，离不开省级机关各部门和苏州等兄弟市的鼎力相助，离不开市人大、政协和社会各界的监督支持，离不开全市人民的团结一心、奋力拼搏。在此，我代表市人民政府，向奋战在各行各业的全体劳动者，向人大代表、政协委员、各民主党派、工商联和无党派人士、各人民团体，向离退休老同志，向中央和省驻宿单位以及人民解放军、武警官兵、公安干警，向参与宿迁建设的海内外客商、高层次人才、广大援宿干部，以及所有关心支持宿迁发展的各级领导、各界人士，致以崇高的敬意和衷心的感谢！

在充分肯定成绩的同时，我们更加清醒地认识到，工作中仍存在一些困难和问题。主要表现在：一是经济增长基础不够稳固，部分行业企业运行较为困难，趋紧的宏观环境影响了项目推进；二是结构性矛盾比较突出，新兴产业和高新技术产业规模偏小，服务业发展水平不高，科技人才支撑能力较弱；三是中心城市功能不够完善，城乡统筹发展步伐较为缓慢；四是改善民生任务还很艰巨，部分小康指标完成压力较大，惠及民生的各项标准还相对较低；五是政府自身建设亟待加强，社会管理水平还需进一步提高，等等。对这些问题，我们一定高度重视，并认真加以解决。

实现新一年又好又快发展

2012年是党的十八大召开之年，也是推进更大突破、实现全面小康的关键一年。新的一年，压力与动力并存，挑战与机遇交织。世界经济形势总体上仍十分复杂严峻，国际经济环境短期内难以明显好转，发达国家经济发展的压力正加速向发展中国家传导；我国经济运行中不稳定不确定因素有所增加，土地、资金、劳动力等要素制约愈加凸显，趋紧的宏观政策环境仍将持续并成为一种常态，给我们保持平稳较快发展带来很大影响。我们既要足够估计面临的困难和挑战，也要充分看到有利条件和积极因素。我国经济发展长期向好的基本面没有改变，中央改善国内经济发展环境的政策取向十分明确，省委省政府支持宿迁发展实现更大突破的力度只会加大不会减弱，尤其是经过长期实践的磨砺，全市上下已形成改革创新的锐气、跨越赶超的志气、敢于拼搏的勇气，这是迎战困难、夺取更大胜利的制胜法宝。

今年政府工作的总体要求是：认真贯彻科学发展观，全面落实市委的决策部署，紧紧围绕“一三六七”的发展思路，坚定不移地扩大有效投入、加快转型升级、统筹城乡发展、增进民生幸福，奋力开创科学发展、跨越发展新局面。主要预期目标是：地区生产总值增长13%左右，财政一般预算收入增长25%，固定资产投资增长25%，规模以上工业增加值增长20%，社会消费品零售总额增长18%，进出口总额增长25%，实际到账外资增长30%，城镇居民人均可支配收入、农民人均纯收入增长14%，节能减排确保完成年度目标任务。这些指标的安排，充分考虑了国内外经济发展走势，充分考虑了立足当前与兼顾长远，充分考虑了发展需要与现实可能，有利于保持平稳较快发展的良好势头。

实现上述目标，需牢牢把握“稳中求进、进中求好、好中求快”的工作导向。“稳中求进”，就是在保持经济运行平稳、物价总体稳定、社会安定和谐的基础上，继续抓住和用好当前重要战略机遇期，努力在经济发展上取得新进展，在改革开放上取得新突破，在民生改善上取得新成效。“进中求好”，就是在推动经济平稳较快发展、社会建设全面进步的同时，更加注重内涵与质量、全面与协调、生态与和谐，加快从外延扩张型增长向内涵提升型增长转变，实现速度、结构、质量、效益相统一。“好中求快”，就是在毫不放松发展质量的前提下，牢固树立“快人一拍、先人一步”的理念，做到措施快落实、项目快推进、目标快完成，始终保持一个相对较快的速度，推动宿迁发展后发快进、跨越赶超。

　　新的一年，我们将着力做好八个方面的工作：

一、狠抓投入扩总量，培育经济增长新动力

牢固树立抓投入就是抓发展的理念，坚持扩大投资规模、提高投资效益两手抓。

全面推进重大项目。今年，全市共安排实施重大项目和中心城市建设项目318个，总投资2086.2亿元，年度计划投资842.2亿元。这些项目如期建成后，城市配套功能将进一步完善，交通路网体系将进一步健全，综合经济实力也将进一步提高。对由政府投资建设的市第一人民医院、重点交通基础设施等项目，建立融资责任机制，落实平台公司融资、土地出让收益、财政性资金安排等渠道筹资责任，为项目推进提供可靠资金保障。对由社会投资建设的运河文化城、可成科技二期、苏酒酒业园区等项目，强化服务保障，力求能快则快，争取早日达效或完成更多的投资额。对其他没有列入重大项目建设计划的项目，也将积极创造条件，加快推进步伐，确保完成建设任务。

全力引进重大项目。在坚定不移推进在手项目的同时，着眼长远发展，谋划运作好重大项目的引进和争取。眼睛向外引项目。抢抓产业转移、企业重组的市场机遇，大力吸引国资、民资、外资等各类投入，对有投资意向的促其快签约，已签约的促其快开工，已开工的促其快推进，尽快形成实际投资量。眼睛向上争项目。紧紧抓住省委省政府支持宿迁发展实现更大突破的政策机遇，全力争取中央和省交通、能源、工业等重大项目布局宿迁，重点加大对徐宿淮铁路、燃气热电厂、国电二期、林浆纸产业园、PTA等重大项目的争取力度，力争年内取得新的突破。

全身心服务重大项目。坚持主动服务、超前服务、贴身服务，努力在趋紧的大环境下创造有利于宿迁投资的小环境。加强项目调度。健全市领导联系挂钩和项目各方主体联动推进机制，积极协调各类生产要素供给，有效化解项目推进和生产经营中的困难。提升帮办水平。认真落实驻厂帮办、检查申报等措施，不断提高为投资主体和项目建设服务的实效性。严格督查推进。对项目建设实行逐月通报、半年考核、年终评比，以鲜明的考核导向和奖惩措施，把全市上下的主要精力集中到项目推进上来。

二、调整结构促转型，推动工业经济新跨越

坚定不移地走新型工业化道路，以产业集聚为目标，以转型升级为主线，加快提升产业集聚度和科技支撑力。

改造升级传统产业。坚持把传统产业作为产业转型升级的重点，加速向技术高新化、管理现代化、品牌高端化方向发展。抓技改促升级。实施“千企升级”行动计划，突出抓好100个5000万元以上省市重点技术改造工业项目，年内完成技改投资360亿元，增长22%。鼓励和支持企业引进新技术、新装备，推动整个产业扩规模、上水平，年内酿酒食品、纺织服装、林木加工、机械电子四大百亿级产业实现销售收入1200亿元，增长40%。抓融合促升级。以20个市级重点企业“两化融合”项目为载体，推动信息技术在企业研发、设计、生产、销售和管理等环节的应用，不断提升传统产业现代化管理水平。抓品牌促升级。实施质量强市战略，年内争创省著名商标6件、省名牌产品15个，支持“双星”、“新箭鹿”等商标创建中国驰名商标。

发展壮大新兴产业。坚持把发展战略性新兴产业作为抢占产业制高点的战略选择，培育壮大新兴产业集群，年内力争规模以上新兴产业销售收入突破450亿元，增长40%。聚焦重点产业。支持新能源、新材料等产业加速突破，鼓励发展生物技术及新医药、节能环保等产业，全年新开工战略性新兴产业项目50个以上。培育领军企业。落实好专项引导资金，促进各类要素向新兴产业优势企业集中，加快培育双星新材、北斗星通、金田塑业、元大科技、欧亚薄膜、荣马新能源、斯迪克新材料等一批有核心技术、有市场前景的领军企业。建设特色园区。聚合力量支持新兴产业基地建设，推动新兴产业在空间、资源等方面集约集聚发展，年内争取建成国家膜材料高新技术特色产业基地。

强化科技创新驱动。坚持把创新驱动作为核心战略，以创新引领产业提升，以人才支撑产业发展。强化科技平台建设。推进苏北工业技术研究院建设，统筹运作好宿迁设施园艺、泗阳杨木加工利用技术、沭阳工业设计与创意等研究院，继续与相关高校合作建立产业技术研究院。突出企业主体地位。鼓励企业加强载体建设，年内新建企业研发机构100个以上、省级工程技术研究中心10个以上，力争规模以上工业企业研发机构建设率达55%。大力发展高新技术产业，年内新增高新技术企业15户以上，实现高新技术产业产值150亿元。注重创新人才引进。深入实施创业创新领军人才“千人计划”及省智力支持宿迁“双百工程”，重点引进和培养高层次人才、紧缺急需人才。加大研发投入力度。年内市、县（区）科技拨款占本级财政支出比例分别达3%和2%，全社会研发经费支出占地区生产总值比重达1.1%。

三、强农惠民助增收，开创三农工作新局面

以农业增效、农民增收为核心，以创建“省级现代生态农业示范市”为抓手，加快农业现代化发展步伐，对农民倾注更深的感情，对农业提供更多的支持，对农村给予更大的倾斜。

大力发展现代农业。推进农业产业园区建设，完善科技培训、种苗繁育、质量检测、电子商务等配套服务平台，年内确保新建省级园2个、市级园3个以上。加快农产品加工集中区建设，推动农业龙头企业扩量提质，重点支持绿港农业、苏北花卉、中粮肉食、益客食品、蒙牛乳业等企业做大做强。加强农业科技创新体系建设，加大新技术、新品种、新模式引进和推广力度，不断增强农业科技创新能力。高度重视农产品质量品牌建设，积极发展生态循环农业，培育壮大特色主导产业。健全完善农产品市场流通体系，市区重点支持华东农业大市场等龙头型市场，优化功能定位，加大产销对接，有序推进农贸市场改造提升；各县集中改造提升或发展一批特色专业批发市场。精心组织好第二届生态农产品展销会。充分发挥江苏农民培训学院的作用，加强新型农村实用人才培养。加快推进农村土地向农村经营大户、农村合作经济组织、农业龙头企业集中，鼓励各种主体兴办各类农业专业化服务组织，年内创建“五好”示范社100家，新增适度规模经营面积45万亩。

改善生产生活条件。加强农田水利建设，实施皂河、来龙等大型灌区续建改造工程，加快农村河道疏浚整治，提高防洪抗旱减灾能力，新增有效灌溉面积5万亩、旱涝保收田12万亩。推进农村沼气、饮水等基础设施建设，年内新建大中型养殖场沼气治理工程15个，新解决35万人饮水安全问题。加大农业综合开发力度，建设高标准农田20万亩。积极推广水稻机插秧技术，不断提高农业机械化水平。深入开展“美好宿迁建设行动”，继续抓好“六整治、六提升”和“三整治、一保障”，年内完成2231个村庄整治，创建省环境优美乡镇10个。

促进农民持续增收。认真落实农民就业创业扶持政策，年内开展农业实用技术培训20万人、农民创业培训1万人，新转移农村劳动力4.5万人。全面落实各种惠农补贴，扩大政策性农业保险范围。继续实施“三来一加”工程，支持农民有效增加经营性收入。推进多种形式的农业适度规模经营，完善多方合作利益联结机制。深化农村土地使用制度改革，全面完成农村土地承包经营权确权登记工作，强化村级“四有一责”建设，稳妥推进“双置换”试点，切实保障农民土地承包经营权、宅基地使用权、集体收益分配权等财产性权利。按照新的扶贫标准确定帮扶农户和经济薄弱村，深入实施西南岗、成子湖周边等贫困地区集中连片开发，加快贫困农户脱贫奔小康和经济薄弱村集体经济增收步伐。加大节能减排力度。坚持把节能减排作为转变经济发展方式的重要抓手，倒逼推进结构调整，加快促进转型升级。节能方面，严把项目节能评估关和环保准入关，加快淘汰落后产能，深入实施40个重点节能和工业循环经济项目，确保年度节能10万吨标准煤以上。减排方面，认真落实结构、工程、管理三大减排措施，健全完善污水收集处理系统，年内所有已建成投运的污水处理厂管网全部配套到位；全面落实重点企业、行业和区域减排责任，加快宿豫污水处理厂二期、宿城耿车污水处理厂以及污水管网配套工程建设，年内新增污水日处理能力19.25万吨。

四、突出重点创品牌，谋求现代服务业新突破

坚持生产性服务业和生活性服务业协同并进，深入实施服务业提速计划，推动服务业发展提速、比重提高、结构提升。

培育壮大旅游业。以开展“旅游投入年”活动为抓手，大力实施旅游业加速突破“三年行动计划”。打造重点景区。加快环骆马湖旅游开发，推进运河文化城、项王故里、皂河古镇等重点景区建设，年内确保项王故里、皂河古镇的核心景区及运河文化创意产业园等项目基本建成；启动建设酒文化广场、博物馆，全力打造洋河工业旅游专线；加快洪泽湖湿地公园创建国家5A级旅游景区和乾隆行宫、项王故里、沭阳花木生态旅游区等创建国家4A级旅游景区步伐。完善服务配套。围绕提升旅游接待服务能力，年内运河金陵、开元名都、华夏丽景等高星级酒店建成运营，力争创建五星级旅游饭店2家、三星级以下旅游饭店10家以上，三星级旅行社5家；年内推出旅游特色商品20种以上。加强营销推介。实施旅游一体化发展战略，统一对外营销推介，打响宿迁旅游品牌，力争全年接待国内外游客800万人次，实现旅游总收入70亿元。

加快发展物流业。以降低物流成本为核心，以打造公铁水联运的现代物流业枢纽城市为目标，推动物流业规模壮大、质态提升。精心编制“十二五”物流业发展规划，明确物流业发展方向，引领全市物流业跨越发展。研究出台扶持物流业发展政策，降低准入门槛，整合现有资源，增强承载能力，减少运营成本；鼓励工业企业主辅分离，支持有条件的大型工业企业组建物流联合体。加快“宿迁物流网”门户网站平台、宿迁中心港、通连物流园等重点物流项目建设，年内物流业增加值占地区生产总值比重提高到7%，物流总费用占地区生产总值比重降低到19%。

做强做精软件和服务外包业。瞄准“苏北软件和服务外包产业排头兵”定位，以招引国内外领军企业和培训机构为重点，突破发展文化创意和行业软件两大智力密集型产业，年内新增软件和服务外包企业80户。继续加大迪信通科技、德俊数码、明俊信息等重点项目推进力度，规划并力争成立苏北工业创意和设计中心，推动京东商城、华军软件、西楚动漫等骨干企业做大做强。年内软件和服务外包产业主营业务收入突破30亿元，离岸外包合同额突破900万美元。

五、完善功能提品位，塑造美好城乡新形象

围绕新型城镇化的总体要求，加快实施中心城市总投资813.6亿元、当年计划投资306亿元的151项重点基础设施建设项目，推动中心城市年年有突破、三年大变样。

强化规划统筹引领。完善城市规划体系。立足提升形象、生态宜居，加快推进第三轮城市总体规划修编，年内完成最终成果报审，并在此基础上完成近期建设规划。立足优化功能、打造亮点，切实抓好中心城市综合交通、绿地系统、文化设施、商业网点等专项规划编制工作，加快推进古黄河水景公园北侧地块、宿豫中心商务区、宿城北片区、宿迁经济开发区核心区、洋河新城核心启动区等重点城市片区设计。立足城乡统筹、协调并进，上半年完成各县城镇体系规划和农民集中居住区规划调整。科学定位组团功能。围绕构建功能各异、协调联动的格局，老城区借助旧城改造，建设集商贸、文娱等功能于一体的传统商业休闲中心；市府新区以发展大道和洪泽湖路为轴线，培育全市的行政中心、现代综合服务中心；宿豫区依托现有基础，建成相对独立、功能配套较为完善的综合片区；宿城新区以南海路为轴线，建设区级行政文化中心及大型公共服务设施；宿迁经济开发区整合区域内各工业园区资源，形成各具特色、分区协同的产业联动格局；湖滨新城发挥滨湖优势，兴建以休闲度假、总部经济、科研教育等功能为主的科技旅游新城；洋河新城结合区域交通条件的提升，打造以酿酒为特色、厂城共建为特征的中心城市副中心和现代化新城区。加大城市建设统筹力度，高标准推进重点片区的资源整合和功能开发，集聚集中建设组团核心区；全面加强组团之间的快速联系，积极探索在旅游、医疗支付等领域推广使用“同城卡”，促进中心城市各组团“内聚外融”。

加快功能提升步伐。完善区域交通体系。加快推进公铁水等多种运输方式建设，主动对接并利用好周边高铁站、港口及机场，逐步建立布局合理、有机衔接的综合交通运输体系。以205国道建设打通市域东部的国家主干线通道、以宿新高速公路建设打通连接连云港的高速出海通道、以245省道建设打通连接三县的县际快速连接通道，全面实现“三通”；加快建设宿新高速公路北互通连接线、250省道至盐徐高速公路宿迁西互通连接线和宿迁中心港连接线，着力推进344省道、发展大道南延至洋河工程和环城西路北延项目，实现中心城市各核心区域与国省干线公路、港口的互连互通，加快推进“六连”；推进宿迁（洋河）火车站、宿迁中心港和公交调度中心建设，并以宿迁（洋河）火车站客货运枢纽为中心，规划建设集“城际客运、城市客运、城乡客运、镇村客运”为一体和集“物流基地、运输服务、信息平台”为一体的客运、货运“两体系”。提升市区商业质态。重点推进苏宁广场、雨润广场、水韵城、中豪国际商业中心等城市综合体项目建设，加快形成以古黄河滨水核心区商圈、霸王举鼎商圈和幸福路商圈组成的市级商业中心为主体、片区级商业中心为骨架、社区公共服务设施为节点的市区商业格局。继续实施中心城市防洪、第二水厂配套、洋河新城供水等工程，加快推进妇产医院、宿迁学院附属学校等社会事业项目建设；启动霸王举鼎商圈地下人防工程和千鸟园广场等应急疏散场所建设，年内幸福路转盘地下人防工程实现主体完工。

六、改革开放添活力，构筑体制机制新优势

以实施全省区域协调发展综合改革试点为契机，创新理念保障发展要素供给，创新举措推动开放型经济突破，创新环境引领民营经济发展，再造宿迁新一轮发展优势。

保障要素供给。在保障项目建设用地上，以推进城乡统筹土地管理创新实验区建设为抓手，探索在全市范围内统筹调剂使用建设用地指标，创新新增建设用地指标市场交易、有偿使用等制度，通过合理评估用地成本，节约集约使用土地；通过合理配置土地要素，保必须、保重点；通过合理引导多元资本，形成整理土地、创造土地指标的整体合力。在保障企业发展资金上，争取浦发银行、浙江稠州银行、苏州银行在宿设立分行，支持交通银行到县区设立支行、苏州在宿银行机构和村镇银行到重点乡镇设立分支机构；推动金融机构向上争取信贷规模，进一步扩大社会融资总规模，年内力争贷款余额突破千亿元；发挥创业投资基金作用，支持初创期科技型企业加快发展；引导和规范民间投融资，探索建立民间借贷阳光机制；改革应急互助基金运作模式，扩大基金规模；严格落实银行信贷有序退出机制，保证企业资金链良性循环。加快企业上市步伐，支持企业利用股权融资、企业债、短期融资券、中期票据、中小企业集合债等方式拓展融资渠道，年内力争直接融资规模超过20亿元。在保障规模企业用工上，健全完善重点企业用工保障机制，成立市、县职业教育技能培训集团，推行订单招工、订单培养、订单服务的“三订”措施，帮助销售收入达亿元、固定资产投资超亿元的“双亿”企业及时解决用工问题，叫响宿迁用工服务品牌。

突破开放型经济。以举办中国（宿迁）第二届经贸洽谈会为抓手，坚持一手抓内资集聚，一手抓外资突破，确保全年引进亿元以上合同项目400个、新开工150个、新竣工100个，竣工项目完成固定资产投资400亿元；实际到账外资增长30%以上。打造台资集聚新高地。以台湾本土及珠三角、长三角等台资集聚区为重点，利用“台湾•江苏周”等平台，加强与各地台协会联系，经常性地组织赴台招商，形成深耕台湾、猛攻台资的强劲势头。坚持“主攻港台、突破日韩、拓展欧美”战略，全力推动外资引进大突破，年内力争新增外资企业100户。提高园区承载力。按照“功能完善、先进适用”的要求，加快推进信息、市场、法规、配套、物流、资金、技术、人才、服务等新的“九通”，建好邻里中心和公共科技平台、投融资平台、人才培训平台“一中心三平台”。高标准规划建设多层标准厂房，提高土地使用效益。全力做好开发区“三创”工作，年内力争宿迁经济开发区创成国家级开发区、苏宿工业园区创成省级开发区，综合保税区创建工作取得重大进展。推动外贸总量再扩大。鼓励企业参加各类境外展会，全方位参与国际经济交流与合作。发挥海关、国检作用，提高对外贸易水平，全年力争实现进出口23.5亿美元。

支持民营经济发展。支持骨干企业做大做强。创造性地落实好现有扶持政策，支持双星新材、秀强股份等重点骨干企业通过强强联合、资产重组、品牌联盟等形式，实现裂变式发展，年内力争新增销售收入亿元以上企业20户，其中双星新材突破50亿元。帮助微小企业发展壮大。对市场前景广、发展潜力大、成长性好的微小企业，专门制定针对性扶持政策，引导其树立远大的发展目标，专注于把主业做精做大，年内新增规模以上企业200户。促进全民创业深入开展。坚持把全民创业放在更加突出的位置，鼓励和引导私营企业做大做强，支持服务业私营企业开展连锁经营，对新开办的个体工商户免收市权范围内管理类、登记类和证照类行政事业性收费，年内新增私营企业3000户、个体工商户3万户。集聚展示城市形象。统筹推进“四城同创”工作，年底前江苏人居环境奖创建达到要求，国家卫生城市创建各项指标达到标准并向上申报，国家环保模范城市和节水型城市创建工作按计划向前推进。围绕形成“一街一景、一路一特色”的目标，推进市区重要道路整体街景的城市设计和整治提升，年内力争打造一批样板街、示范路和精品区。大力实施中心城市水系沟通工程，加快西沙河、古黄河、西民便河疏浚整治，年内完成古黄河雄壮河湾南岸景区建设，让古黄河成为流动的河、清澈的河、美丽的河、繁荣的河。高标准建设生态廊道系统，年内市区新增绿地12万平方米。加强城市日常管理，加快数字化城管建设，深入开展户外广告、占道经营等专项整治行动，实施中心城市出入口美化工程，努力扮靓城市形象。下决心推进城乡生活垃圾收运处置体系建设，年内市区生活垃圾分类处理和资源化利用率达10%以上，主次干道机械化清扫率达40%。

七、提升内涵重均衡，实现社会事业新发展

加快社会事业发展步伐，不断提高公共服务质量，更好地满足人民群众日益增长的精神文化需求。

推动文化事业大繁荣。完善重点文化服务设施，启动市图书馆改造提升工程，完成市博物馆新馆建设。结合运河文化城建设，统筹实施市科技馆、广播电视发射塔等项目建设。规划实施好城市“15分钟文化圈”、农村“十里文化圈”建设。继续抓好文化“三乐”、“三送”工程，年内送戏500场、送电影1.5万场、送书10万册以上。新增有线电视用户10万户。发挥文艺作品“楚风奖”导向作用，创作一批文化精品力作。推进“文化产业发展年”活动，加快“三带”、“三园”、“六区”等文化产业集聚区建设，支持报业传媒集团、广电集团等骨干企业做大做强，年内文化产业增加值增长30%以上。以创建省级文明城市为抓手，广泛开展群众性精神文明创建活动，提高全社会文明程度。注重发挥文化引领和塑造作用，丰富提升城市文化内涵，增强市民归属感和自豪感。

推动教育事业创品牌。推进教育现代化建设，不断提升教育发展内涵。加快义务教育均衡发展，逐步缩小区域、城乡和校际差距。调整优化市区教育布局，以优质学校为龙头实施集团化办学。深入实施学前教育普及提高和优质创建工程，加快提升普通高中教学质量和办学水平，年内创建省优质幼儿园20所、省三星级以上高中2所。探索校企合作办学机制，年内所有公办中等职业学校全部建成四星级以上学校，中等职业学校毕业生本地就业率超过40%。加快高等教育发展，支持宿迁学院深化办学体制改革，提高办学水平。认真落实扶持政策，促进民办学校健康发展。

推动卫生事业上水平。继续对接实施基本药物制度，在现行试点的基础上，逐步向全市所有一级医疗机构推开，年内基本药物全部实行限价销售，并通过延伸结报网络等措施，方便老百姓就医和报销。按照三级甲等医院标准，加快推进市第一人民医院建设，同步实施人才招聘、引进和培训工作。积极支持市人民医院创建三甲医院、沭阳人民医院创建三级医院。全面加强乡村医疗卫生机构建设，鼓励全市所有二级综合医院与乡镇医院建立合作关系，推行乡镇卫生院和村卫生室一体化管理，逐步提高农村医疗水平。

八、改善民生增和谐，满足人民群众新期盼

始终把惠民生、增和谐作为政府工作的根本追求，加快实施居民收入“六年倍增”计划，推进公共服务“六大体系”建设，努力优化民生生态，今年财政用于民生投入比上年增长30%以上。

办好民生实事项目。围绕学有所教，年内新建和加固校舍45万平方米，所有乡镇中心校运动场实施塑胶化改造，为各县区幼儿园免费定向培养100名五年制专科层次幼儿教师。围绕住有所居，年内实施危旧片区和棚户区改造150万平方米，新开工经济适用房5150套，新增廉租房620套、公租房2980套，建设拆迁安置房185万平方米。围绕病有所医，年内开展孕前免费健康检查试点，实施贫困儿童大重病医疗救助，对纳入范围的城乡低保对象及其他特困群体实施医疗救助。围绕劳有所得，年内城镇新增就业2.5万人、下岗失业人员再就业1万人，组织城乡劳动力技能培训3万人。围绕老有所养，年内建成126个居家养老服务中心，新增“关爱工程”床位650张，农村五保老人集中供养率达65%，五保集中和分散供养标准提高到每人每年4000元和3200元，80岁以上老年人全部发放尊老金。围绕弱有所助，年内城乡低保标准提高到每人每月270元和240元；免费为1000名贫困白内障患者实施复明手术、1000名残疾儿童进行康复训练、1000名残疾人实施就业培训，帮助1000名残疾人实现就业梦想。围绕食有所安，年内持续开展食品药品安全专项整治，大型餐饮单位和学校食堂100%安装运行监管系统；新增平价蔬菜商店30家以上。围绕行有所便，年内完成374公里农村公路和69座桥梁建设改造，新增或更新城乡公交车150辆，新辟或优化城市公交线路10条，行政村客运班车通达率达100%，实现农村居民一次换乘到县城、二次到市区。

健全社会保障体系。大力推进城镇居民基本养老保险工作，全面实施医疗保险、生育保险和失业保险市级统筹。强化扩面征缴，年内“五险”基金收入达19.5亿元。城乡居民养老保险实现适龄人员参保和60岁以上老年居民领取基础养老金“两个全覆盖”。落实各项社会保障待遇调整政策，稳定提高基本养老、医疗保险待遇标准。城镇居民医保参保率、新农合参合率稳定在98%以上，新农合筹资标准提高到年人均300元，城镇职工、居民医保和新农合政策范围内医疗费用报销比例分别达85%、70%和70%。创新医疗救助运行机制，推进城乡医疗救助同步结算平台建设。完善特殊困难群众生活救助物价上涨动态补贴机制，提升基层社会救助规范化管理水平。大力发展社会福利事业，加快构建社会养老服务体系，落实孤儿基本生活保障制度，促进慈善公益事业健康发展。推进公共就业服务体系建设，重点抓好就业援助政策落实，积极帮扶高校毕业生、农村劳动力、困难群体和退役士兵就业。

加强和创新社会管理。探索建立“三社联动”机制，重点抓好社区用房和居民公益性服务设施建设，逐步推行“一委一居一站一办”服务管理模式，全年新增达标社区191个。加大对重要领域、重点行业和重大危险源的监控力度，强化事故隐患排查治理，坚决遏制重特大事故发生。全面推行校车安全标准，建立健全长效管理机制，保障学生出行安全。切实加强应急管理，努力提升突发事件应对和处置工作水平。完善矛盾纠纷排查调处机制，对社会矛盾和问题多发领域进行集中排查、集中整治，全力维护社会稳定。深入推进平安宿迁、法治宿迁建设，加快构建城乡一体的社会治安大防控格局，不断增强群众的安全感和满意度。推动各项事业协调发展。夯实计划生育基层基础管理，稳定低生育水平。全面实施妇女儿童“十二五”发展规划，提升妇女儿童事业发展水平。继续推进“十分钟健身圈”建设，积极申办全国自行车公开赛，办好全国小轮车锦标赛等赛事活动，组队参加省第六届全民健身运动会。加强国防动员和民兵预备役建设，深化“双拥”共建，进一步密切军政、军民关系。

建设人民满意的服务型政府

推进更大突破、实现全面小康对政府自身建设提出了新的更高要求。我们将牢固树立“政务就是服务”的理念，重点在执政为民、依法行政、提升效能、改进政风上再造政务服务新优势，为全市人民安居乐业和广大投资者创业致富提供优质服务。

坚持以人为本，以执政为民的追求让人民更加满意。始终突出民本。把群众呼声视为工作导向，把群众需求作为服务追求，对事关人民群众的事，再小也是大事；对人民群众要求的事，再难也要抓紧办好。坚决做到凡有面子、没里子的事坚决不干，凡只顾眼前、不顾长远的事坚决不干，凡与民争利、损害民权的事坚决不干。时刻关注民情。组织政府组成部门和广大机关干部深入基层、深入群众，问计于民、问政于民、问需于民，从实践中发现工作推进的问题和不足，从群众中提炼破解难题的经验和做法，从基层中捕捉创新工作的思路和方向。继续开展“群众不满意的事”大征集活动，关注不满意，解决最急需，追求更满意。致力服务民生。对于政府而言，就业富民的目标，没有最高，只有更高；改善民生的追求，没有最好，只有更好。围绕城乡居民收入“六年倍增”目标，多施富民之策，多辟富民之道，让群众的腰包在宿迁跨越发展的进程中更快地鼓起来。坚持从群众最关心、最迫切的问题抓起，重点实施好8大类48项为民办实事项目，不断改善群众生产生活条件，让弱者感觉到生活的温馨，让普通人感觉到生活的幸福。

严格依法行政，以规范透明的制度让人民更加满意。健全决策程序。认真执行市政府工作规则和会议制度，制定出台《宿迁市重大行政决策程序规定》，坚持把公众参与、专家论证、风险评估、合法性审查和集体讨论决定作为必经程序，保障公众在行政决策事前、事中和事后的充分参与。规范执法行为。深入推行行政执法责任制，健全完善行政执法程序。坚持严格执法、公正执法、文明执法，按照法定权限和程序履行职责，进一步规范行政执法自由裁量权，实现行政执法公信力和人民群众满意度的“双提高”。主动接受监督。及时向社会公开政务信息，加强政府门户网站建设，完善网络问政平台，健全市、县（区）新闻发布及信息公开制度，打造网络发言人和政务微博品牌，进一步畅通政民互动渠道。主动接受市人大及其常委会的法律监督，积极支持市政协履行政治协商、民主监督和参政议政职能。认真办好人大代表议案、建议、意见和政协委员提案，让政府工作更加契合时代要求、更加符合群众意愿。

提升工作效能，以勤勉尽责的服务让人民更加满意。提高行政审批效率。着眼于简政放权、转变职能，深入推进行政审批“三集中、三到位”改革，对现行所有的市级行政审批事项继续进行清理、调整和压缩，做到应减必减、该放就放。全面落实首问负责、服务承诺、限时办结等制度，在实践中不断形成有利于推动工作的理念、目标和方法，进一步提高服务质量和水平。上半年市“一办三中心”以全新的服务内容、服务机制和服务面貌进入“便民方舟”运行，加快构建更具宿迁独特竞争力的服务品牌。完善工作推进机制。强化“今天再晚也是早、明天再早也是晚”的意识，积极推行条线工作法、一线工作法，对重要工作采取周抽查、旬调度、月通报、季总结的工作机制，环环紧扣抓推进，毫不松劲抓落实。健全督查考核体系。对人代会及其常委会、政府相关会议形成的决策意见，对重大项目、重点工程和为民办实事项目，实行全覆盖、全方位、全过程督查，确保政令畅通、落实到位。

大力改进政风，以清正廉洁的形象让人民更加满意。加强廉政建设。全面落实党风廉政建设责任制，扎实推进惩治和预防腐败体系建设。积极倡导良好的生活作风，培养高尚的道德情操。坚持勤俭办事。牢固树立长期过紧日子的思想，反对铺张浪费，严控行政成本，集中财力保证重点支出。对已批准的在建项目和维修改造项目，实行财政投资评审，按照批复概算组织实施。强化监督考核。加强政府内部廉政监察、执法监察、效能监察和责任审计，抓好重大工程、重点行业、重点领域和重要环节的监管，坚决制止行政不作为、乱作为、慢作为，努力塑造风清气正的良好环境。

各位代表，在“十二五”发展征程上阔步前行的宿迁，正焕发着前所未有的勃勃生机。让我们在省委、省政府和市委的正确领导下，解放思想，坚定信心，团结拼搏，务实苦干，奋力开创全市经济社会又好又快发展的新局面，为推进更大突破、实现全面小康而努力奋斗！
